# Can time-series of Landsat data be used to map the onset of growth at 78°N (central Svalbard)?

**DOI:** 10.1007/s00484-026-03276-4

**Published:** 2026-07-22

**Authors:** Laura Stendardi, Giovanni Argenti, Luigi Ranghetti, Stein Rune Karlsen

**Affiliations:** 1 Department of Agriculture, Food, Environment, and Forestry (DAGRI), University of Florence, Piazzale delle Cascine, 18, Florence, FI 50144 Italy; 2 Bergamo, Italy; 3 NORCE Norwegian Research Centre AS, P.O. Box 6434, Tromsø, N-9294 Norway

**Keywords:** Phenology, Landsat 8, Svalbard, NDVI, Arctic vegetation

## Abstract

Mapping vegetation phenology in the high Arctic is critical for understanding ecosystem responses to rapid warming, but persistent cloud cover and low solar angles pose substantial challenges for optical satellite monitoring. We evaluated whether Landsat 8 can successfully map the start of growing season (SOS) at 78°N in Svalbard by developing spectral-based cloud detection algorithms tailored to Arctic conditions. From 17 images during the 2014 growing season, we generated cloud-free NDVI time series and extracted SOS dates at 30-m resolution across Adventdalen (Advent Valley). Validation against six field plots equipped with phenocameras demonstrated strong agreement (R² = 0.884, RMSE = 4.1 days, NSE = 0.869). The resulting SOS map revealed systematic spatial patterns ranging from late May to early August, with elevation explaining 6.6% of variance as the strongest single predictor. Combined topographic models (elevation, slope, distance from sea) explained 14.6% of variance, indicating that unmeasured factors particularly aspect, snow distribution, and vegetation composition exert dominant controls on phenological timing. These results confirm the viability of Landsat-based phenology mapping in the high Arctic. Critically, by establishing a validated processing framework this study provides the methodological foundation for application to the historical Landsat archive (Landsat 4/5 TM and Landsat 7 ETM+), subject to cross-sensor radiometric calibration. This opens unprecedented opportunities for reconstructing 30-m resolution phenological time series extending back to 1984, enabling quantification of four decades of vegetation responses to Arctic warming in regions experiencing some of Earth’s most rapid climate change.

**Supplementary Information:**

The online version contains supplementary material available at 10.1007/s00484-026-03276-4.

## Highlights

First application of Landsat 8 for Arctic phenology mapping at 78°N achieves strong field validation (R² = 0.884).

Growing season onset spans 75 days (late May to early August) across fine-scale topographic gradients.

Topographic controls (elevation, slope, distance from sea) explain only 14.6% of phenological variance.

High spatial heterogeneity reveals complex interactions between microclimate, snow, and vegetation composition.

Opens 40-year Landsat archive for medium-resolution phenology before Sentinel-2 era.

**Supplementary Information:**

The online version contains supplementary material available at 10.1007/s00484-026-03276-4.

## Introduction

The timing in phenology phases, defined as the science of the timing recurring biological events (Lieth 1974), is affected by ongoing environmental changes (Piao et al. 2019). Warming is occurring in the Arctic faster than anywhere else on the planet (Intergovernmental Panel on Climate Change 2014; Previdi et al. 2021); for this reason, investigating the response of Arctic tundra to climate change is a challenging topic. Existing literature already assessed that the structure and composition of Arctic vegetation have changed, causing both an increase in primary production and an alteration in ecosystem properties (Sturm et al. 2001; Chapin et al. 1995; Rixen et al. 2022). The Svalbard archipelago lies in the High Arctic and an increase in mean annual air temperature of 3–5 °C was observed from 1971 to 2017 (with winter warming reaching 5–8 °C), accompanied by a decrease in the length of the snow season (Hanssen-Bauer et al. 2019). Since these variables influence the vegetation life cycle (Wielgolaski 1999; Cleland et al. 2007; Callaghan et al. 2011), the study of vegetation phenology is critical to understanding the consequences of climate change in the archipelago. Notably, changes in the start of the growing season can have a strong impact on these ecosystems. Different timing of spring activity affects the distribution and abundance of Arctic animals, such as the arrival of migratory birds and reindeer population dynamics (Aanes et al. 2000; Walther et al. 2002; Jensen et al. 2008).

Satellite remote sensing images can be used in the analysis of vegetation growing season. However, validation of satellite products using field data is often complex at these latitudes. The limitations include the inaccessibility of the locations, the costs for the personnel collecting the data in the field, and the narrow temporal window for sampling imposed by the brevity of the Arctic growing season (Lillesand et al. 2015).

Optical satellite data like MODIS (Karlsen et al. 2014) and Sentinel-2 (Karlsen et al. 2021) were successfully used to map the start of the growing season of the Svalbard vegetation. These studies highlighted the complexity of obtaining cloud-free time series due to cloud cover on the one hand and the low solar angle on the other hand, which limits the application of cloud masks (Park et al. 2016; Vickers et al. 2020; Karlsen et al. 2021). Furthermore, while MODIS achieves a spatial resolution of a maximum of 250 m pixels (Karlsen et al. 2014), Sentinel-2 allows investigating the start of the growing season in much greater detail, albeit only from 2016 onwards (Karlsen et al. 2021). Exploiting all available optical imagery is therefore crucial, both to obtain medium-resolution data prior to Sentinel-2’s operational period and to enable multi-scale integration across different sensors. In this context, the Landsat program, which has acquired imagery since 1972, provides an exceptional opportunity for studying Arctic phenology. Landsat 4, launched in July 1982, collected data until late 1993, while Landsat 5, launched in 1984, operated for nearly 29 years, one of the longest-running Earth observation missions. The subsequent launches of Landsat 7, 8, and 9 have extended this archive to over four decades, with many years benefiting from overlapping coverage from multiple Landsat satellites. While Landsat satellites (4–9) have a 16-day repeat cycle at the equator, their near-polar orbits provide a key advantage for Arctic studies: at central Svalbard (78°N), overlapping orbital paths result in much more frequent image acquisition, enabling denser temporal sampling of the brief growing season. Imagery from Landsat 8 OLI, operational since February 2013, provides crucial medium-resolution (10–30 m) data for phenological studies. While the 2015 launch of Sentinel-2 A enabled harmonized Landsat-Sentinel-2 (HLS) products with multiple daily acquisitions at high latitudes, the 2014 season represents the last complete pre-Sentinel-2 year. At Longyearbyen (central Svalbard, 78°N), Landsat 8’s near-polar orbit delivered exceptional coverage: 117 images over the 152-day growing season (1 May to 30 September 2014), representing a 77% daily acquisition rate, an unprecedented temporal density for medium-resolution optical data.

The overall objective of the study is to develop cloud-free Landsat 8 time series that can provide relevant data on the phenology of Arctic vegetation and in detail:

1) to derive spatially-explicit information on the start of the growing season (SOS) at medium spatial resolution (30 m), validating satellite-derived estimates through field-collected phenological data;

2) to apply an established SOS detection methodology to Landsat 8 medium-resolution data, supporting multiscale and multi-sensor integration for Arctic phenological monitoring, while analyzing the relationships between derived SOS estimates and topographic variables to quantify fine-scale environmental controls on growing season onset.

Although only one year (2014) is studied, the results demonstrate the potential for extending the approach to the full historical Landsat archive back to 1984. Landsat 8 serves as a methodological reference by providing the processing framework which, subject to cross-calibration between Landsat 8 OLI and the previous Landsat 4/5 TM and 7 ETM+ sensors, can be applied to reconstruct medium-resolution (30 m) phenological time series covering four decades of Arctic warming (Isaksen et al. 2022).

## Materials and methods

### Study area

**Fig. 1:**
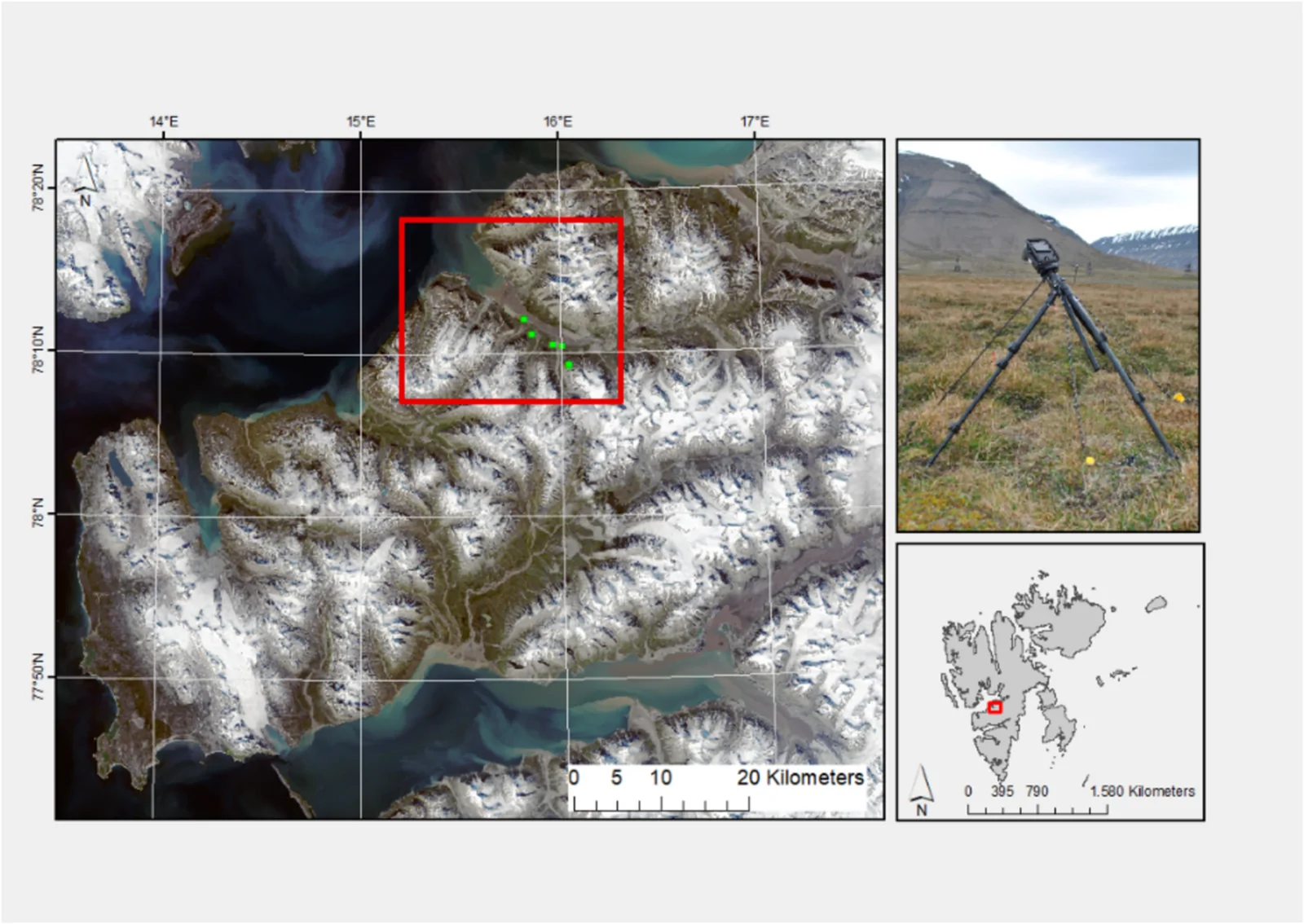
Study area - The main figure shows a Landsat-8 RGB composition (LC8-4) of the study area highlighted in red, and the field site location displayed in green; on the right panel an image of a field camera and a map of the Svalbard archipelago with the study area outlined

Our study area is the Adventdalen (Advent Valley) and surrounding mountain plateaus located in the central part of the island of Spitsbergen (Fig. 1). The area lies approximately between latitudes 78⁰ 20’ and 78⁰ 07’ N, and longitudes 15⁰ 10’ and 17⁰10’ E. River Adventelva flows through the valley, shaping a floodplain and a flat terrace of silty soil.

The Adventdalen is in the mid-Arctic tundra zone according to bioclimatic criteria. The surrounding mountain plateaus reach the northern Arctic tundra zone and even the Polar desert (Elvebakk 1985). Annual total precipitation is low, about 200 mm, and soil has a high content of mineral components. The vegetation in the valley has a mosaic character, depending on whether it develops on the ridges or in the wetlands. On the ridges the *Dryas octopetala* L. community is prevalent, where species such as *Salix polaris* Wahlenb., *Bistorta vivipara* (L.) Delarbre and *Saxifraga oppositifolia* L. are common. On ridge tops and slopes *Cassiope tetragona* (L.) D.Don often dominates. In lowland areas, mossy tundra with dominants of *Aulacomnium turgidum* (Wahlenb.) Schwägr and *Tomentypnum nitens* (Hedw.) Loeske is present. Wetlands are dominated by bogs, fens and mossy tundra, depending on the water table.

### Datasets

#### Landsat 8 data

We investigated a Landsat 8 dataset covering the 2014 vegetation growing season (May–September), acquired from the Earth Resources Observation and Science (EROS) Center of the United States Geological Survey (USGS). The year 2014 was selected as it represents the first complete growing season archive available, since the 2013 season fell within the satellite’s initial on-orbit checkout and calibration period. The study area is covered by WRS-2 paths 22–29 and 211–218, and rows 3–5 and 240–241. During the 2014 growing season, 117 Landsat 8 images were available for the study area.

**Fig. 2:**
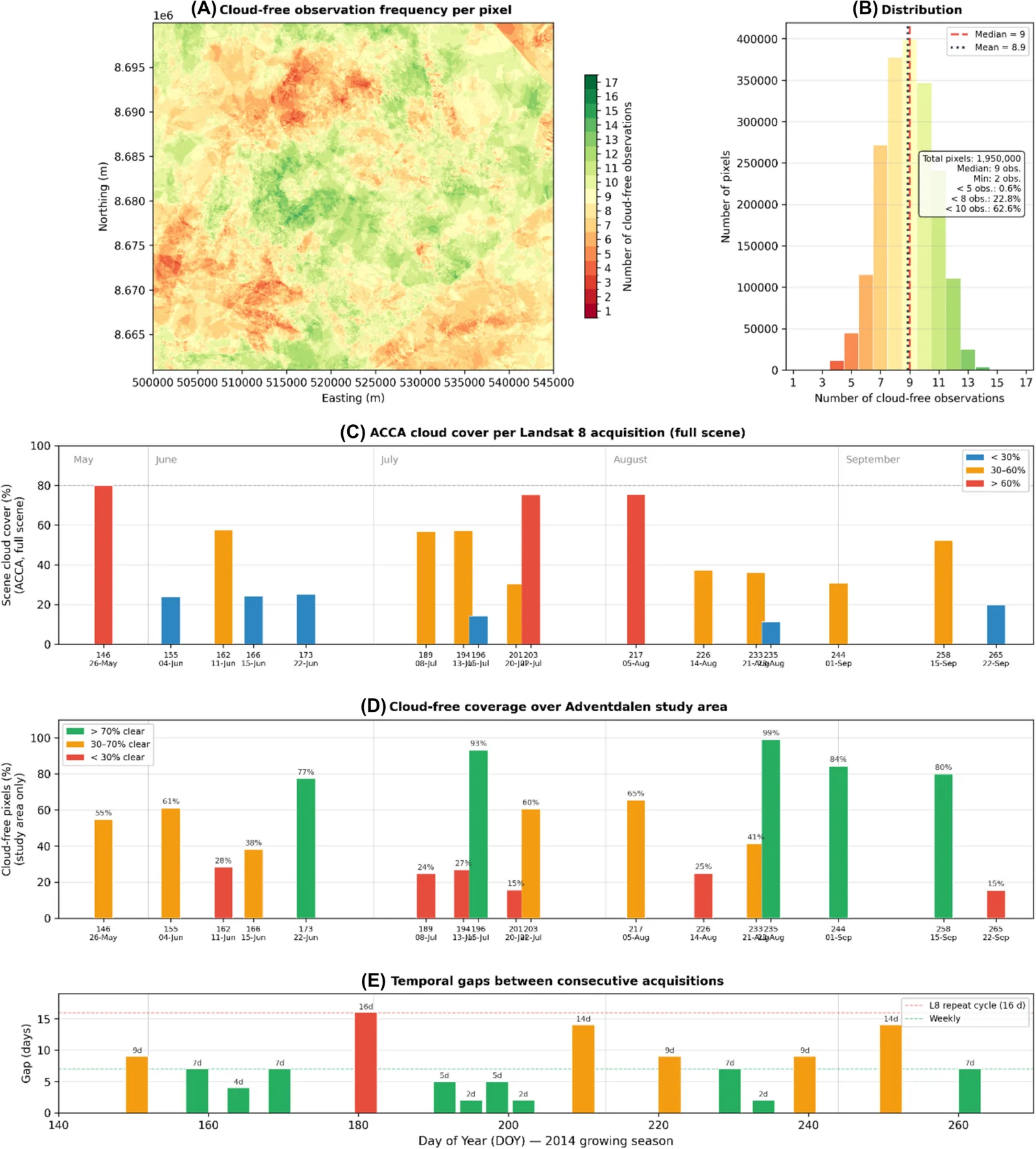
Data availability for the 17 usable Landsat 8 acquisitions during the 2014 growing season at Adventdalen. (**A**) Per-pixel cloud-free observation frequency; the colour scale ranges from red (2 observations) to green (16 observations). (**B**) Distribution of cloud-free observation counts, with the vertical dashed line marking the median (9 observations). (**C**) ACCA scene-level cloud cover for the full Landsat scene. (**D**) Cloud-free pixel coverage restricted to the study area. (**E**) Temporal gaps between consecutive acquisitions; dashed lines mark the Landsat 8 repeat cycle (16 days) and a weekly reference

From this archive, we applied a cloud cover threshold of < 80% based on the Automated Cloud-Cover Assessment (ACCA) algorithm (Irish et al. 2006) to discard heavily cloud-covered scenes. All remaining candidates were then visually inspected for cloud and shadow coverage specifically over the Adventdalen study area. Of the 117 available scenes, only 17 contained any cloud-free pixels over the area of interest; the remainder were entirely cloud-covered over the study area and therefore unusable. ACCA-reported cloud cover for the 17 usable scenes ranged from 11% to 80% (Table 1), though actual cloud-free coverage over the study area often differed substantially from the scene-level estimate (Fig. 2, Panels C-D). The 17 usable scenes provided a mean temporal revisit of 7.4 days (median 7.0 days), but with substantial irregularity: gaps between consecutive acquisitions ranged from 2 to 16 days depending on cloud conditions (Fig. 2, Panel E). Cloud-free observation frequency also varied considerably across the study area, from a minimum of 2 to a maximum of 16 observations per pixel (median 9). Indeed, 62.6% of pixels were observed fewer than 10 times over the entire growing season, and individual scenes contributed cloud-free data for as little as 15.3% (DOY 265) up to 98.8% (DOY 235) of the study area (Fig. 2, Panels A–B).

**Table 1:** List of Landsat 8 images used in our study. The table shows the dates expressed in days and DOY, the identification code of each image, and the cloud cover in percent according to the ACCA algorithm

Code	ID	Day	DOY	Cloud cover (%)
LC8-1	LC82160042014146LGN00	26-MAY-14	146	79.97
LC8-2	LC82150042014155LGN00	04-JUN-14	155	23.89
LC8-3	LC82160042014162LGN00	11-JUN-14	162	57.48
LC8-4	LC80272402014166LGN00	15-JUN-14	166	24.06
LC8-5	LC80282402014173LGN00	22-JUN-14	173	25.16
LC8-6	LC80282402014189LGN00	08-JUL-14	189	56.55
LC8-7	LC82160032014194LGN00	13-JUL-14	194	57.09
LC8-8	LC82140042014196LGN00	15-JUL-14	196	14.15
LC8-9	LC82170032014201LGN00	20-JUL-14	201	30.23
LC8-10	LC82150042014203LGN00	22-JUL-14	203	75.16
LC8-11	LC82170042014217LGN00	05-AUG-14	217	75.46
LC8-12	LC82160042014226LGN00	14-AUG-14	226	37.03
LC8-13	LC82170032014233LGN00	21-AUG-14	233	36.14
LC8-14	LC82150042014235LGN00	23-AUG-14	235	11.29
LC8-15	LC82140042014244LGN00	01-SEP-14	244	30.55
LC8-16	LC82160042014258LGN00	15-SEP-14	258	52.15
LC8-17	LC82170042014265LGN00	22-SEP-14	265	19.62

#### Phenological in-situ data

Ground-truth phenological observations were collected at seven monitoring sites across Adventdalen during the 2014 growing season as part of the Environmental Monitoring of Svalbard and Jan Mayen program (MOSJ, www.mosj.no/en). Sites were selected to represent the major vegetation types of the study area, including *Dryas octopetala* dominated ridges, *Cassiope tetragona* heath, *Salix polaris* snowbeds, and mossy tundra communities (Table 2). Field plots were located along the accessible side of the valley, following the road connecting Longyearbyen to the mining infrastructure; the opposite side of the valley was not sampled due to the absence of safe access routes, the risk of polar bear encounters away from monitored areas, and the limited time available for fieldwork given the high logistical costs of Arctic campaigns. As a result, plots span an elevation range of 7–489 m a.s.l. but a comparatively narrow range of slope aspects (mostly east- to southeast-facing, shifting to north-facing at the two lowest, coastal-proximal sites; Table 2), a constraint we revisit when interpreting the topographic controls on SOS.

**Table 2:** Description of ground stations location (UTM zone 33 north, datum WGS84) and their attributes

Name	ID	Coordinates	Elevation (m a.s.l.)	Aspect	Description
Kjell Henriksen Observatory	P01	523,625 E, 8,675,237 N	489	127° (SE)	The plot is characterized by a scattered vegetation with *Salix polaris*, *Saxifraga nivalis*, *Stellaria longipes* Goldie and *Oreomecon dahliana subsp. polaris (Tolm.)* Elvebakk & Bjerke. Situated 15 m east of the road.
EISCAT	P02	523,812 E, 8,675,773 N	424	89° (E)	Situated north-east of EISCAT Svalbard Radar, the plot is characterized by *Salix polaris* and *Luzula confusa* Lindeb. plant community. *Stellaria longipes*, *Oreomecon dahliana* and *Alopecurus ovatus* Knapp. are also common.
Gruve 7	P03	523,776 E, 8,675,873 N	417	77° (E)	The area is characterized by scattered moss-rock snowbed community, with *Salix polaris*, *Alopecurus ovatus* and *Ranunculus sulphureus* Sol., situated 10 m east of the road.
MAB-station	P04	523,020 E, 8,677,964 N	29	78° (E)	The site is in an alluvial fan with *Salix polaris*, *Bistorta vivipara*, *Alopecurus ovatus* and *Eriophorum scheuchzeri* Hoppe subsp. *arcticum* M.S.Novos.
Tredalshytta	P05	521,913 E, 8,678,107 N	32	70° (E)	The plot is characterized by a *Cassiope tetragona* heath, with *Salix polaris*, *Oxyria digyna* (L.) Hill, *Stellaria longipes* and *Dryas octopetala*.
Svalbardhytta	P06	519,621 E, 8,679,368 N	34	62° (NE)	Situated about 10 m from the road, the plot is characterized by *Salix polaris*, *Dryas octopetala*, *Silene acaulis*, *Bistorta vivipara* and *Cassiope tetragona*.
Dammyra	P07	518,720 E, 8,681,011 N	7	4° (N)	Situated about 25 m east of a side road, the plot shows a *Salix polaris* snow bed in transition to dry moss tundra.

Time-lapse cameras (Acorn LtL-5310WA, 12-Megapixel, 100° wide angle) were installed at each site on tripods approximately 50 cm above the ground surface, capturing hourly images between 10:00 and 14:00 throughout the growing season. Each camera monitored approximately 1 m² of vegetation.

The SOS was determined through manual analysis of phenocamera images following the extended BBCH phenological scale. The BBCH scale (Biologische Bundesanstalt, Bundessortenamt und CHemische Industrie) provides a standardized system for coding phenologically similar growth stages across plant species. We defined SOS as the date when the monitored species reached BBCH stage 15, corresponding to “>5 leaves unfolded, but not yet full size” for shrubs (primarily *Salix polaris*), and “>5 leaves (> 3 cm) clearly visible” for graminoids ((Poaceae, Cyperaceae, and Juncaceae) (Karlsen et al. 2021). This phenophase occurs rapidly in Arctic environments and represents the onset of clearly visible vegetation green-up, making it suitable for validation of satellite-derived phenological metrics. We acknowledge that *Saxifraga nivalis* L., and *Silene acaulis* (L.) Jacq are evergreen species for which flowering represents their most easily recognized phenophase. These species are not necessarily the most suitable choices for phenological monitoring but were included in Table 2 primarily as indicators of the characteristic vegetation type of each plot.

### Methodology

**Fig. 3:**
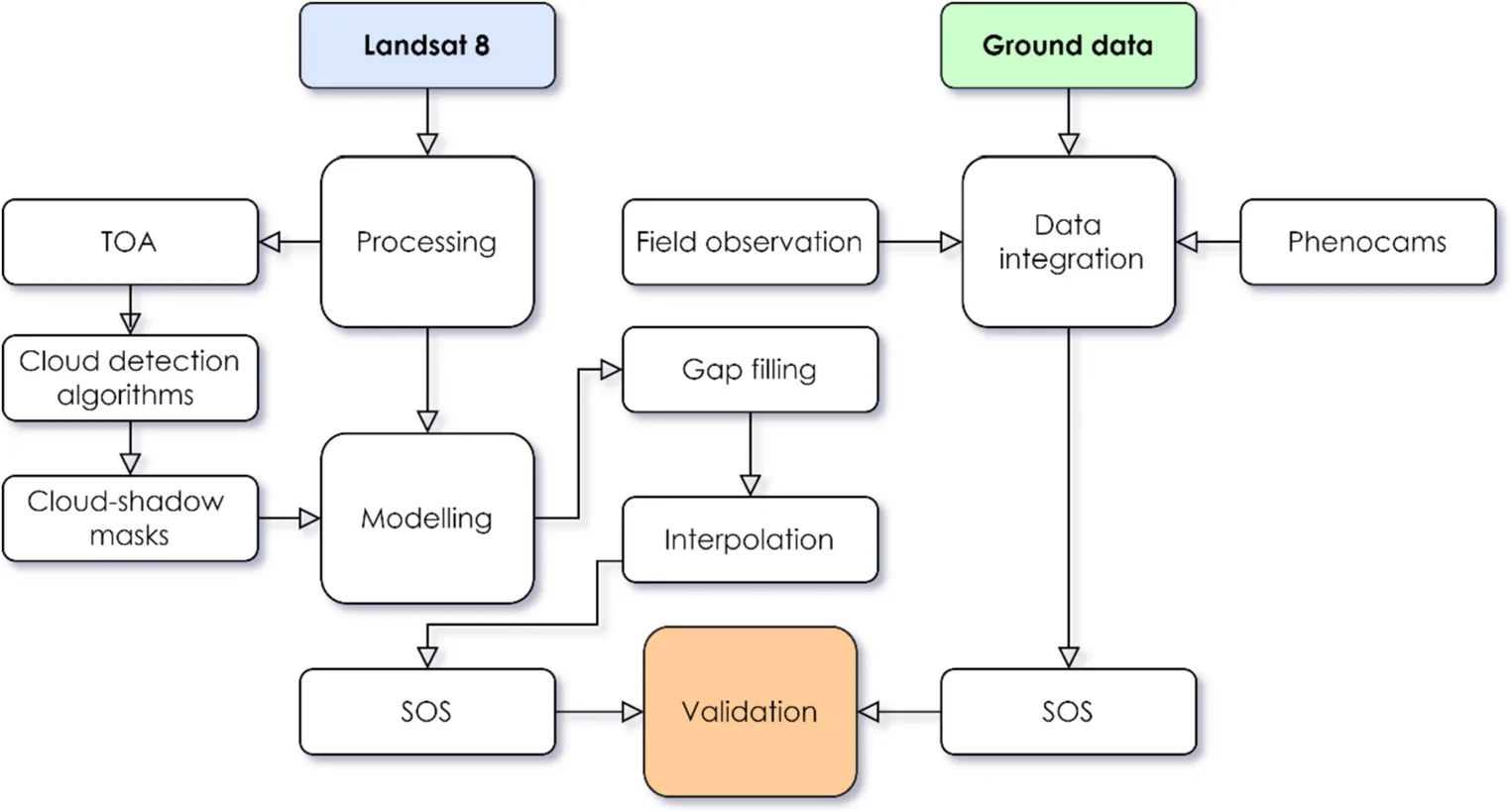
The figure illustrates the methodology of the current work

This study followed a multi-stage research workflow, as illustrated in Fig. 3.

#### Data modelling and validation

The Landsat 8 images were downloaded as Level-1TP (Terrain Precision Corrected) products from the USGS EROS Center, which are already radiometrically calibrated and orthorectified as standard. Then, TOA conversion of the time series was performed using Exelis ENVI v.5.3. Each optical band of the images was rescaled to Top Of Atmosphere (TOA) reflectance (bands 1–9) and radiance (bands 10–11). Subsequently, the atmospheric contributions of the radiance data in the thermal infrared were removed from the TIRS bands. Emissivity and temperature information were split in the radiance data, obtaining the surface temperature in Kelvin. Since high latitude implies low solar elevation angles (solar zenith angles greater than 70°), which vary considerably during the summer season, and due to the similar reflectance signatures between snow/ice surfaces and clouds, commonly used cloud masks ( Irish et al. 2006; Zhu and Woodcock 2012) do not allow for a full detection of cloud-covered pixels. Therefore, it was required to develop ad hoc algorithms to define clouds and their shadows across the entire time series. A combination of algorithms was associated with each image to generate two layers for detecting clouds and their shadows. The algorithms thresholds were determined empirically through iterative visual inspection of the scenes, guided by the known spectral properties of clouds and snow/ice surfaces at high latitudes (Gao et al. 1993; Irish 2000; Irish et al. 2006). Therefore, six algorithms were created for a cloud mask and three algorithms for cloud shadows. A list with a description of the algorithms used and their application to the Landsat 8 images is provided in the Table 3.

**Table 3:** Algorithms developed for the definition of surfaces covered by clouds and their shadows. The table lists the algorithms and the images to which they were applied

ID	Algorithm (based on L-8 bands)	Surface	L-8 images
CL-1	Band 7 > 0.17 OR Band 9 > 0.53	Cloud	LC8-1, LC8-3, LC8-5, LC8-6, LC8-17
CL-2	Band 7 > 0.13 OR Band 10 < 276 AND Band 10 > 273	Cloud	LC8-2
Cl-3	Band 9 > 0.020	Cloud	LC8-12, LC8-13
CL-4	Band 1 < 0.36 AND Band 1 > 0.20 OR Band 7 > 0.28	Cloud	LC8-11
CL-5	Band 7 > 0.22 OR QA > 38,784	Cloud	LC8-4, LC8-7, LC8-8, LC8-9, LC8-10, LC8-14, LC8-15
CL-6	Band 9 > 0.623 OR QA > 45,056	Cloud	LC8-16
SH-1	Band 7 < 0.03	Shadow from cloud	LC8-1, LC8-17
SH-2	Band 5 < 0.07	Shadow from cloud	LC8-2, LC8-3, LC8-4, LC8-5, LC8-6, LC8-11, LC8-12, LC8-13, LC8-16
SH-3	QA = 20,480	Shadow from cloud	LC8-7, LC8-8, LC8-9, LC8-10, LC8-14, LC8-15

**Fig. 4:**
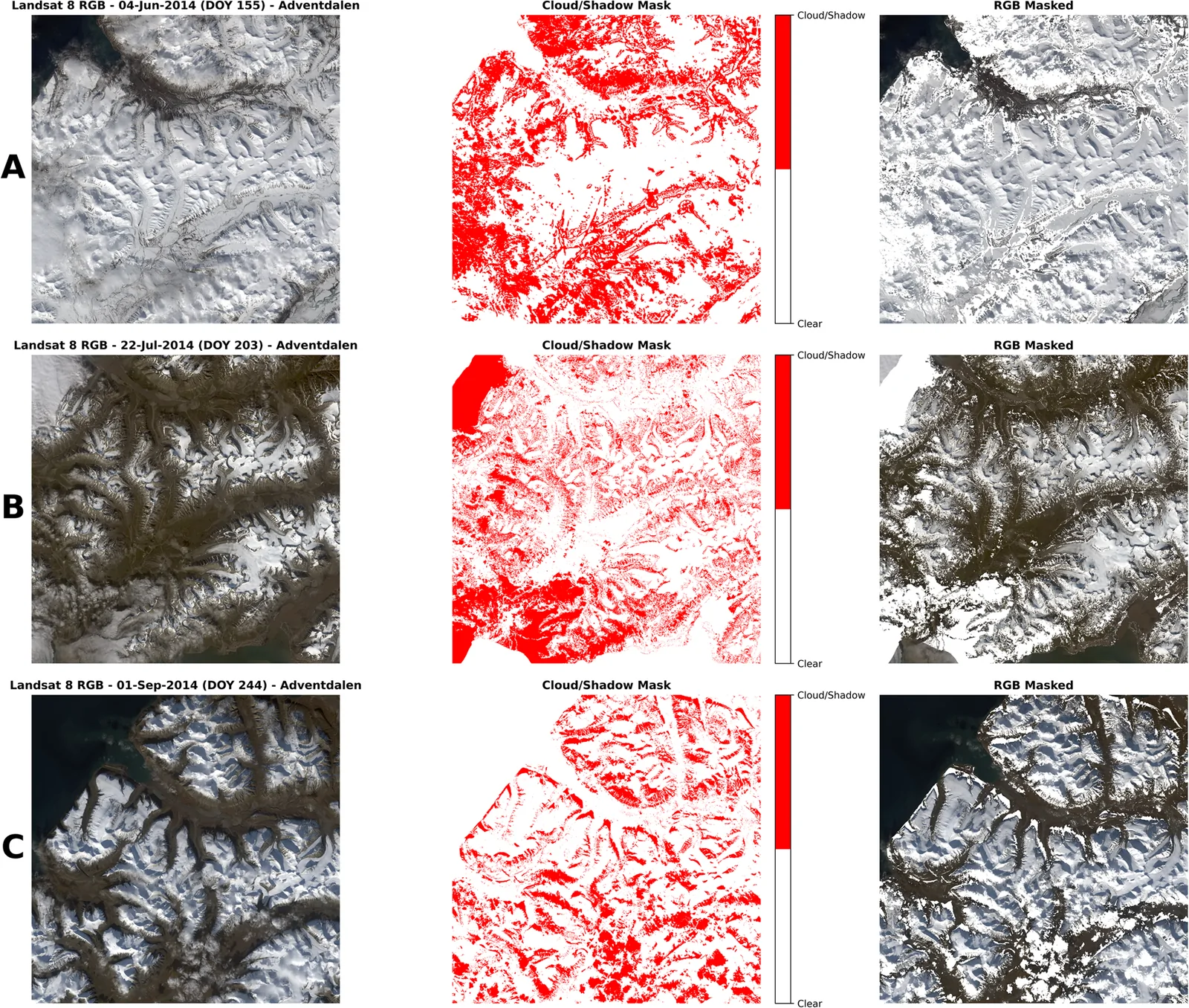
Cloud and shadow masking results for Adventdalen, Svalbard. (**A**) 4 June 2014 (DOY 155, 24% clouds), (**B**) 22 July 2014 (DOY 203, 75% clouds), (**C**) 1 September 2014 (DOY 244, 31% clouds)

Figure 4 illustrates the performance of the cloud and shadow masking algorithms for three dates representing low (4 June, 24%), high (22 July, 75%), and moderate (1 September, 31%) cloud cover conditions. Water bodies (sea, rivers, and lakes) were also masked. Despite low solar elevation angles at 78°N, the algorithms effectively discriminated between clear and cloud-contaminated pixels across all conditions.

Then, each couple of cloud and shadow masks were combined to obtain the masks that were used to extract cloud-free images for all optical bands in the 2014 dataset. Next, using a weighted average based on the time intervals (Formula 1.1), the cloud pixels were filled with the resulting values.

1 $$\begin{array}{c}Value\;(t1)=(Value\;(t0)\times(t2-t1)\\-Value\;(t2)\times(t0-t1))/(t2-t0)\end{array}$$

Where t1 is the timestamp of the image to be filled, while t0 and t2 are the timestamps of the closest preceding and subsequent images, respectively.

Finally, linear interpolation was applied to the 17 images by developing a new dataset containing 119 daily images from DOY 146 to DOY 264, from which the Normalized Difference Vegetation Index (NDVI) was calculated and smoothed using a Savitzky–Golay filter with a window size of 5 and 2 iterations.

To link surface phenology with NDVI values, we calculated for each pixel the average NDVI value for the period July 12 to August 5, following Karlsen et al. (2014), who identified this interval as corresponding to the phase of maximum vegetation greenness in the Adventdalen area. NDVI averaged values during the phase of maximum vegetation were used to weight NDVI daily maps in order to reduce the ‘noise’ from snow-covered terrain (Karlsen et al. 2014, 2021). The SOS in each pixel was then defined by the time when each day’s NDVI value exceeded 0.70 of the average value, following the threshold established by Karlsen et al. (2014) for the same study area.

To investigate environmental controls on SOS timing, we extracted elevation and slope from a 20 m spatial resolution Digital Elevation Model (DEM) of the study area. Slope was calculated using Horn’s method (Horn 1981) with a 3 × 3 weighted kernel. Distance from sea was computed using Euclidean distance transformation on the sea mask. Ordinary least squares linear regression models were fitted to explore relationships between SOS and three topographic variables: elevation, slope, and distance from sea. Variables were standardized prior to analysis to allow comparison of effect sizes. An additive model and a set of models including two-way interaction terms (Elevation×Distance, Elevation×Slope, Slope×Distance) were compared to test for context-dependent effects of topography on SOS timing.

Concerning the field data, the SOS of each plot was estimated both by using the information collected directly from the observers and by visually analysing the camera images. In this case, we defined the SOS as the date of general flowering of catkins of *Salix polaris*. This species is among the most common vascular species on Svalbard, occurs in most habitats, and the flowering is easily recorded and occurs only a few days after the leaves are unfolded. Hence, from a distance this is often seen as a greening of the vegetation, where this species is common. These estimates were validated by comparing them with camera images when available. Conversely, from July onwards we only used the information coming from the cameras. Camera-based phenological monitoring provides several advantages: minimal interference with permanent plots, consistent temporal coverage, and objective documentation of vegetation development. However, manual interpretation of camera images introduces potential subjectivity, particularly given the small size of Arctic plant leaves and reproductive structures. The rapid onset of the growing season in Svalbard (typically occurring within a few days once initiated) helps minimize temporal uncertainty in SOS determination. Afterwards, a simple linear regression was fitted between satellite-derived and field-observed SOS dates to assess agreement. Validation metrics were computed using Python (scipy, scikit-learn): R² and Pearson correlation coefficient, Root Mean Square Error (RMSE) and Nash-Sutcliffe Model Efficiency (NSE).

## Results and discussion

### Achieving cloud-free time series

Our spectral-based algorithms proved effective for discriminating clouds and shadows in this high-latitude environment, as demonstrated through visual inspection of the generated masks across scenes representing a range of cloud cover and illumination conditions (Fig. 4). Band 9 (Cirrus: 1.36–1.38 μm) was particularly valuable, successfully detecting both high-altitude cirrus clouds (Gao et al. 1993), and low-level clouds prevalent in polar regions. Band 7 (SWIR2: 2.11–2.29 μm) enabled discrimination between clouds and snow/ice features based on their differential reflectances (Gao et al. 1993). Additional spectral ranges (Band 1, Band 10, QA band) complemented the primary detection algorithms.

### The spatial pattern of the start of the growing season

The SOS map produced for the Adventdalen area shows a spatially coherent distribution of phenological timing, consistent with the known environmental characteristics of the site. The gap-filling and daily interpolation approach adopted here facilitated the reconstruction of the full growing season trajectory, enabling SOS extraction across the study area.

**Fig. 5:**
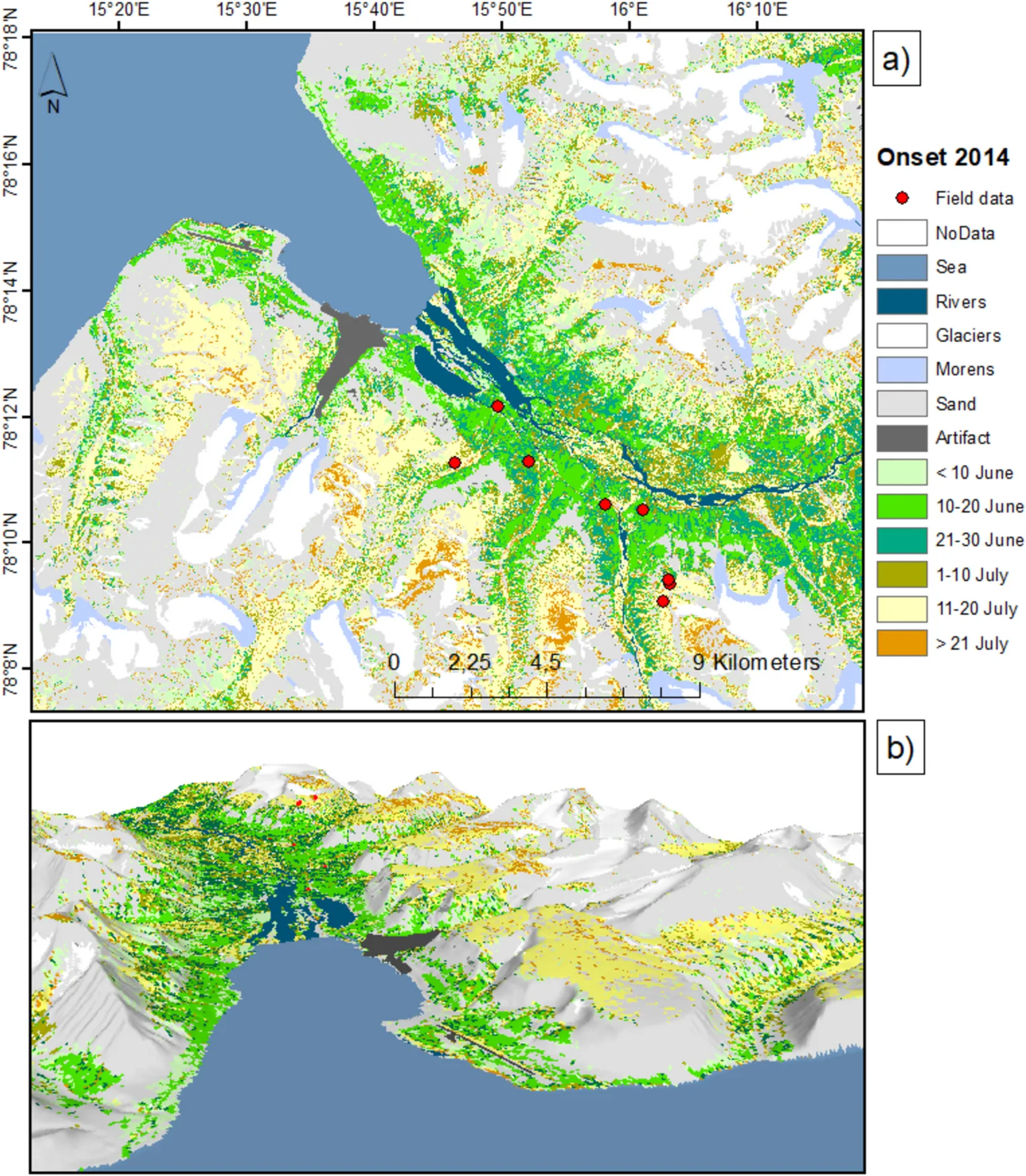
Map (panel **a**) and 3D view (panel **b**) of the SOS in Adventdalen, also showing the position of the field plots

The next steps of time series modelling resulted in a SOS map within our study area (Fig. 5). According to the map in Fig. 5, SOS occurs before 10 June in the floodplain, a few metres above sea level. Between 10 and 20 June, SOS is observed in the S-W part of the floodplain, reaching 150 m above sea level. From 21 to 30 June, SOS occurs at the bottom of the slopes and in the tributary valleys, while from 1 to 10 July it is observed close to the river and at the top of the mountain plateaus, at almost 500 m above sea level. Between 11 and 20th July, SOS occurs on the underside of the ridges and on the slopes, mixed with areas of earlier onset. After 20 July SOS reaches 500 m a.s.l. and is scattered along the sides of the ridges. The mapping showed a wide spatial variability of the SOS influenced by the spatial and elevation gradient as well as the proximity of the river and the sea. This spatial gradient of SOS is consistent with findings from other medium- and low-resolution studies in Svalbard (Karlsen et al. 2014, 2021). It should be noted that the SOS detection approach was anchored to the phenology of the dominant plant functional types in the study area, namely shrubs and graminoids, which were also used as reference species for field validation. While this ensures methodological consistency, vegetation composition inevitably influences the timing and magnitude of the NDVI signal at the pixel level. The aggregation of spectrally and phenologically diverse vegetation types within a single 30 m pixel represents an inherent limitation of the threshold-based approach, and the retrieved SOS should therefore be interpreted as an integrated signal rather than a species-specific estimate. Future studies incorporating vegetation maps could allow a more stratified analysis of phenological patterns across plant communities.

### Environmental controls on SOS timing

To quantify the environmental factors influencing SOS variability, we analyzed the relationships between SOS timing and topographic variables (elevation, slope, distance from sea) for 225,339 pixels across the study area.

The environmental characteristics varied systematically across SOS categories (Table 4). Early-onset areas (< 10 June, DOY < 161) occurred at intermediate mean elevations of 308 ± 225 m with steeper slopes (17.6 ± 11.2°) and relatively close to the sea (5.4 ± 3.6 km). In contrast, late-onset areas (> 21 July, DOY > 201) were found at substantially higher elevations (527 ± 217 m) with moderate slopes (14.1 ± 11.3°) and farther inland (8.1 ± 3.2 km). The intermediate categories showed progressive transitions across these environmental gradients, indicating systematic topographic control on phenological timing.

**Table 4:** Environmental characteristics by SOS category

SOS Category	*N* pixels	%	Elevation (m)	Slope (°)	Distance (km)
< 10 June	33,671	14.9	308 ± 225	17.6 ± 11.2	5.4 ± 3.6
10–20 June	36,602	16.2	120 ± 133	10.4 ± 9.4	5.7 ± 4.4
21–30 June	30,316	13.5	139 ± 145	9.4 ± 9.5	7.3 ± 4.3
1–10 July	33,826	15.0	202 ± 172	11.6 ± 10.0	7.3 ± 4.0
11–20 July	76,593	34.0	357 ± 191	12.5 ± 10.4	6.7 ± 3.8
> 21 July	14,331	6.4	527 ± 217	14.1 ± 11.3	8.1 ± 3.2

Topographic variables showed statistically significant correlations with SOS timing (all *p* < 0.001; Table S1, Panel A). Elevation exhibited the strongest relationship (*r* = 0.257, R² = 0.066), with a gradient of approximately 2.1 days per 100 m elevation gain. Distance from sea showed a positive but weaker correlation (*r* = 0.151, R² = 0.023), corresponding to a delay of 0.66 days per kilometer inland. Slope showed a weak negative correlation (*r* = -0.093), with steeper slopes paradoxically associated with slightly earlier onset. Slope also showed moderate positive correlation with elevation (*r* = 0.402), suggesting complex interactions between topographic variables).

**Fig. 6:**
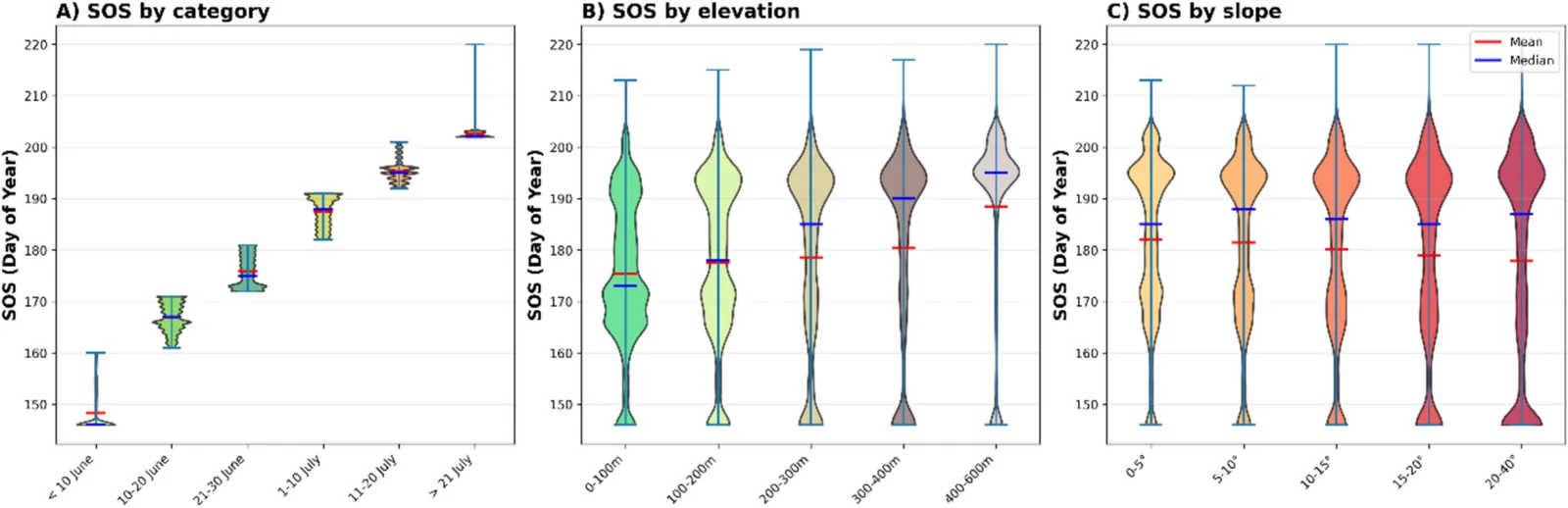
Distribution of start of season (SOS) timing across environmental gradients: (**A**) by SOS category showing temporal progression and variability within each class; (**B**) by elevation bands demonstrating later onset at higher elevations; (**C**) by slope classes. Violin width represents pixel density, with red lines indicating means and blue lines indicating medians. Early-onset areas are characterized by lower elevations and steeper slopes, while late-onset areas occur predominantly at high elevations

In univariate regressions, elevation alone explained 6.6% of SOS variance. A combined additive model including all three topographic variables improved explanatory power to 12.3%. Addition of interaction terms (Elevation×Distance, Elevation×Slope) further increased R² to 14.6%, with conditional effects revealing that the elevation gradient decreased from 3.5 days/100m near the coast to less than 1 day/100m at 15 km inland. Similarly, the elevational effect diminished on steeper slopes, suggesting context-dependent relationships between topography and phenology (Fig. 6).

The modest explanatory power (R² = 0.146, RMSE = 16.3 days) reflects the inherent complexity of Arctic ecosystems, where high spatial heterogeneity in microclimatic conditions creates phenological variability not fully captured by simple topographic variables. Elevation emerged as the strongest predictor (R² = 0.066), reflecting systematic delays in snowmelt and temperature accumulation with increasing altitude. Distance from sea showed a weaker but significant effect (R² = 0.023), with sites farther inland experiencing delayed onset, likely due to the moderating influence of maritime air masses on coastal microclimate and earlier snow disappearance in areas exposed to maritime influence. Aspect (slope orientation) creates substantial phenological differences in Arctic environments, with south-facing slopes experiencing earlier onset than north-facing slopes due to differential solar radiation receipt (Karlsen and Elvebakk 2003). Notably, our field validation plots themselves span a narrow range of aspects (predominantly east- to southeast-facing; Table 2), reflecting the accessibility constraints of the study area. This limits our ability to assess how well the satellite-derived SOS captures aspect-driven phenological variability, and represents a further argument for expanding field validation to additional, more topographically diverse sites in future work. Fine-scale snow distribution patterns, driven by wind redistribution and topographic sheltering, directly control the timing of soil exposure and warming (Inouye and Wielgolaski 2025), with snowmelt date being a primary determinant of growing season onset (Kudo and Hirao 2006). However, snow distribution varies heterogeneously even within 30-m pixels, and its effects on phenology are mediated through complex interactions with subsequent soil moisture and temperature accumulation patterns, as plants at later-melting sites can accelerate development by experiencing higher mean daily temperatures. Vegetation composition differences, ranging from *Dryas octopetala*-dominated ridges to *Cassiope tetragona* heath and mossy tundra in depressions (Karlsen and Elvebakk 2003), introduce phenological variability that reflects complex interactions between microsite conditions, snow accumulation patterns, and soil properties (moisture, organic content, thermal characteristics) not captured by our elevation, slope variables and distance variables. The counterintuitive negative correlation between slope steepness and SOS (steeper slopes = earlier onset) likely reflects confounding effects of improved drainage, reduced snow accumulation on steep terrain, and the prevalence of south-facing aspects among the steepest slopes in our study area. Despite these limitations, the significant relationships between topographic variables and SOS timing demonstrates that Landsat 8 captures meaningful phenological variability across the Arctic landscape, providing valuable information for monitoring vegetation responses to climate change at regional scales.

### Phenological in-situ data

The results are shown in Table 5, where the DOY is associated with the SOS of each plot. Due to camera failure at the Kjell Henriksen Observatory site (P01), ground-truth SOS data were obtained for six of the seven monitoring locations. The earliest onset of growth was recorded in the Tedalshytta plot (DOY 169) at about 30 m a.s.l. One day later (DOY 170), vegetation growth occurred in two plots at the entrance to the valley of the Dammyra and Svabardhytta plots, at an altitude of 10 and 30 m a.s.l., respectively. Then the vegetation started to grow in Mab station, also at 30 m above sea level but further into the valley. Finally, the Gruve7 and EISCAT plots experienced a delayed start to the season, as they were at an elevation of about 410 to 420 m above sea level, respectively.

**Table 5:** Day of the year where the vegetation growing season started in 2014

SOS 2014							
PLOT	Dammyra	Mab station	Svabardhytta	Tredalshytta	Gruve7	EISCAT	Henriksen
DOY	171	182	171	169	196	196	N.a.

**Fig. 7:**
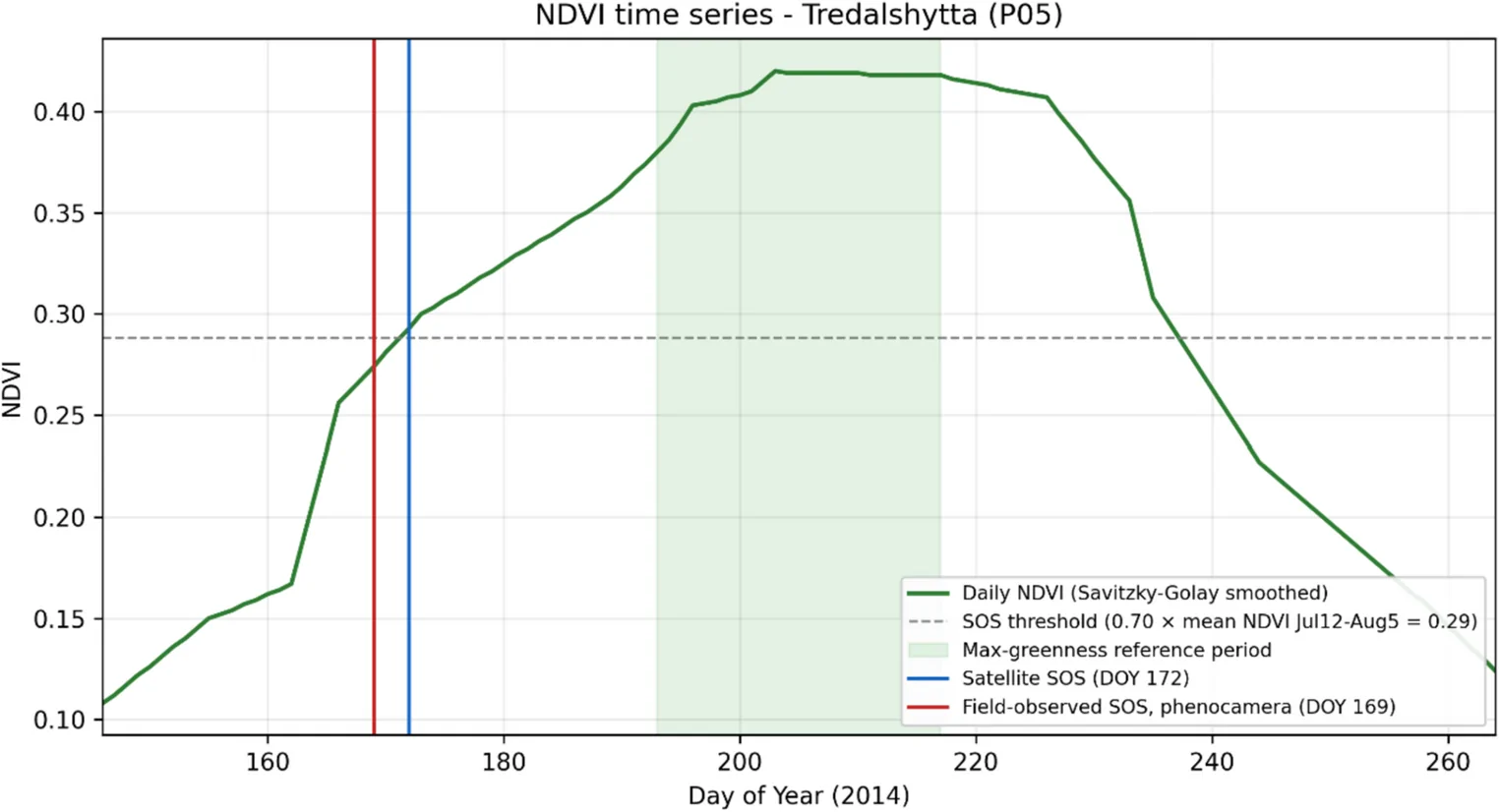
Representative NDVI time series at Tredalshytta (P05), one of the field validation sites, illustrating the SOS detection approach. The shaded band indicates the max-greenness reference period (12 July − 5 August) used to define the SOS threshold (0.70 × mean NDVI of that period, dashed line). The blue and red vertical lines indicate, respectively, the satellite-derived and field-observed SOS dates

Figure 7 illustrates this SOS detection process for a representative site, showing the daily NDVI trajectory, the satellite-derived threshold, and the corresponding field-observed date.

Our visual assessment approach is consistent with methods employed in previous Arctic phenology studies (Anderson et al. 2016), though future applications could benefit from automated image analysis techniques to further reduce observer bias and enable processing of larger image datasets.

### Evaluation of SOS map reliability

**Fig. 8:**
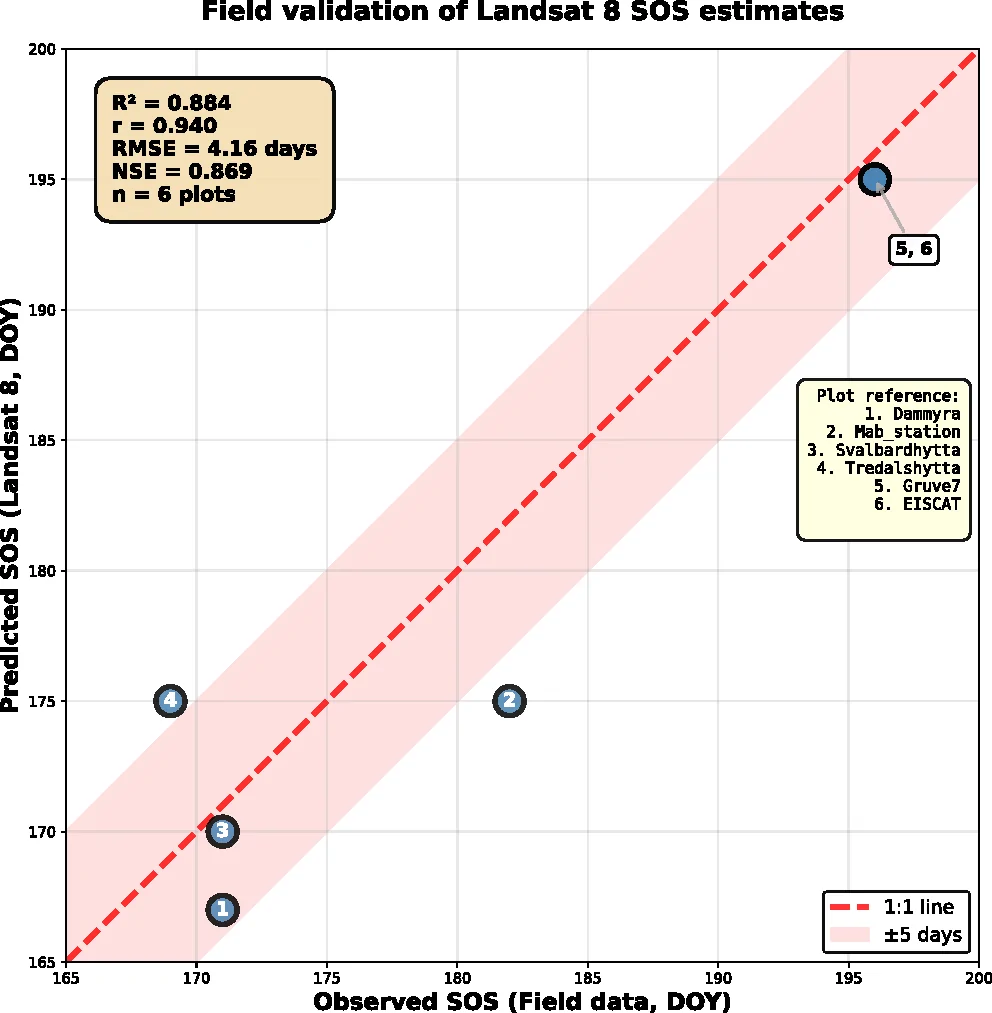
Field validation of Landsat 8 SOS estimates. Each point represents one field plot, with observed values from phenocameras and field observations versus satellite predictions (DOY). The 1:1 line (red dashed) indicates perfect agreement

The map evaluation though field data provided strong results, confirming the validity of SOS estimations and so the reliability of the produced map. The regression between relative SOS estimated from NDVI time-series and from ground truth data ($\:{\mathrm{R}}^{2}$=0.884, *p*= 0.007), underscore the high predictive power of the performed method (Fig. 8). The high correlation between predicted and measured values was also confirmed by the high value of the Pearson sigma coefficient, corresponding to 0.94. The RMSE was equal to 4.1 days, an error which is comparable with the time lag between consecutive satellite acquisition, and for this reason which is considered as an acceptable error. The NSE statistic allowed verifying the efficiency of the predictions, in a range from −∞ (highest error) to 1 (complete correspondence): our model is proved to be equal to 0.869, a high value which confirms the goodness of the estimations.

### Observation density and implications for historical reconstruction

Whether this approach can be extended to the historical Landsat archive depends, above all, on the number and temporal distribution of cloud-free observations available in a given year. Our per-pixel analysis (Fig. 2) shows that even in 2014 when Landsat 8 provided 117 acquisitions over the study area thanks to the convergence of multiple WRS-2 paths at high latitudes the median number of usable observations per pixel was only 9, and over 60% of pixels were observed fewer than 10 times. Notably, individual scenes varied from 15% to nearly 99% cloud-free coverage, highlighting how unevenly clouds affect data availability across the study area. These figures are consistent with the findings of Bayle et al. (2024), who showed that observation density and growing season length together account for most of the variance in sampling-induced biases for satellite-derived vegetation metrics in Alpine environments, conditions that closely parallel those encountered at Svalbard, where growing seasons are short and cloud-free windows are rare.

However, our approach differs from the maximum compositing framework examined by Bayle et al. (2024) in one important respect. Their analysis focused on NDVImax derived from raw Landsat observations, where the probability of capturing the true seasonal peak depends directly on how many scenes are available. We do not extract metrics from raw composites; instead, we first reconstruct continuous daily NDVI profiles through gap-filling and Savitzky-Golay interpolation (Formula 1.1), and then derive SOS from the fitted curve. This type of temporal reconstruction is, in fact, one of the correction strategies that Bayle et al. (2024) recommend for reducing observation-dependent biases. That said, the quality of the reconstructed curve still depends on having enough input observations with adequate temporal spread, as our pixel-level statistics make clear.

For earlier Landsat missions, the actual number of archived scenes at high latitudes is likely to be considerably lower. All Landsat satellites share the same 16-day repeat cycle and orbital convergence at polar latitudes, but acquisition and archival practices differed substantially across missions. During the Landsat 5 era, a failure in the Tracking and Data Relay Satellite System (TDRSS) link meant that data could only be downloaded when the satellite was in direct view of a ground station, and during the commercialisation period images were acquired on demand rather than systematically (Zhang et al. 2022). The introduction of the Long-Term Acquisition Plan with Landsat 7 (Arvidson et al. 2006) brought more systematic global coverage, but the scan-line corrector failure on 31 May 2003 left approximately 22% of pixels missing in every subsequent ETM+ scene (Maxwell et al. 2007), further reducing usable data. It was only with Landsat 8 that near-complete acquisition of all land overpasses became operational practice (Wulder et al. 2016). Developing automated cloud masking procedures suitable for high-latitude scenes, together with a systematic, year-by-year assessment of observation density along the lines recommended by Bayle et al. (2024), will be a necessary step before phenological reconstruction can be reliably extended to the full Landsat archive at 78°N.Where the number of cloud-free observations in a single year remains insufficient, aggregating data across adjacent years (i.e. biennial composites) offers a potential strategy to increase the density of viable observations, at the cost of reduced temporal resolution of individual SOS trends. This approach may prove particularly valuable for extending phenological reconstruction into the earlier, more cloud-limited years of the Landsat archive, and for improving the statistical robustness of long-term SOS trend detection in future climate change assessments.

The overall aim of our study was to obtain information on the start of the growing season in the high Arctic environment. Landsat 8 has proved to be successfully usable in defining the SOS. The validation results compare favourably with previous studies in the same area using different sensors. Karlsen et al. (2014) reported R² = 0.60 (*n* = 25) for MODIS-based SOS against field observations of *Salix polaris* flowering, while Karlsen et al. (2021) obtained R² = 0.47 (*n* = 38) using Sentinel-2 data across seven vegetation types. The higher R² obtained in the present study (0.884) is partly attributable to the smaller and more homogeneous validation dataset (*n* = 6 plots), which limits the generalisability of the comparison. This reflects the data available for the 2014 growing season specifically, for which no additional independent field observations exist beyond the six plots used here. An important direction for future work is therefore to extend the present framework to a multi-year analysis, leveraging field observations available for growing seasons before and after 2014 together with the corresponding Landsat archive, which would allow for a substantially larger and more robust validation dataset. Thus, our study enabled us to obtain a medium-resolution map that could be evaluated alongside the data subsequently generated using higher resolution sensors such as Sentinel-2, or combined with other Landsat missions including Landsat 9, to extend the temporal coverage of phenological monitoring in the high Arctic. Moreover, this result opens important perspectives for the integration of mid-resolution sensors in the high Arctic. The integrated use of more than one sensor (e.g. Harmonized Landsat and Sentinel-2 (HLS) have been increasingly applied to detect vegetation phenology (Bolton et al. 2020; Kosczor et al. 2022). As discussed above, persistent cloud cover at 78°N severely limits the number of usable observations even with the highest-density modern archives, and this constraint will need to be carefully assessed for each target year when working with historical missions (Shen et al. 2021; Bayle et al. 2024). Multi-sensor integration approaches for Arctic phenology thus necessitate labor-intensive preprocessing steps to mitigate persistent cloud-related data gaps, a consideration that must be weighed against the benefits of enhanced temporal coverage.

## Conclusion

This study demonstrates that Landsat 8 time series can successfully map the start of the growing season in the high Arctic (78°N), despite the substantial challenges posed by persistent cloud cover and low solar elevation angles. Through development of spectral-based cloud detection algorithms tailored to Arctic conditions, we generated a cloud-free NDVI time series for the 2014 growing season in Adventdalen, Svalbard, enabling extraction of spatially explicit SOS dates at 30-m resolution.

The resulting SOS map revealed systematic spatial patterns strongly consistent with field observations (R² = 0.884, RMSE = 4.1 days, NSE = 0.869), with green-up onset ranging from late May (DOY 146) to late August (DOY 220) across the landscape. Environmental analysis demonstrated that topographic variables (elevation, slope, distance from sea) explained 14.6% of SOS variance, with elevation showing the strongest control (2.1 days delay per 100 m). However, the modest explanatory power indicates that unmeasured factors, particularly aspect, fine-scale snow distribution, and vegetation type composition, likely exert dominant influences on phenological timing in this complex Arctic terrain. Notably, the near-daily acquisition frequency at 78°N due to orbital overlap provides temporal coverage comparable to lower-latitude sites despite the 16-day nominal repeat cycle, representing a significant methodological advantage for phenological monitoring in this region.

These results confirm the viability of applying medium-resolution satellite phenology methods, previously validated with MODIS and Sentinel-2, to Landsat data in high-Arctic environments. Looking ahead, extending this approach to the historical Landsat archive (1984–present) is feasible in principle, but not straightforward. The observation density achievable in each specific year will be the key limiting factor, and automated cloud detection methods that work reliably at high latitudes will need to be developed before the full 40-year Landsat record can be leveraged to quantify vegetation responses to rapid Arctic warming.

Future applications should incorporate aspect analysis and integrate multi-sensor approaches combining the full Landsat family (Landsat 7,8 and 9) with Sentinel-2 (from 2015 onwards) to maximize temporal coverage and observation density. While the cloud-masking procedures developed here were labor-intensive, they provide a template for processing historical Landsat archives in polar regions where standard algorithms often fail. Understanding long-term phenological trends in these rapidly changing ecosystems is critical for assessing climate change impacts on Arctic vegetation, carbon cycling, and wildlife habitat availability. Two priorities emerge for future research. First, extending the reconstruction back to 1984 requires a cross-sensor calibration framework accounting for the spectral and radiometric differences between Landsat 8 OLI and the earlier Landsat 4/5 TM and 7 ETM+ sensors, including the data gaps introduced by the Landsat 7 SLC-off failure from 2003 onwards. Second, a formal sensitivity analysis quantifying how SOS retrieval accuracy degrades with decreasing observation density is necessary to assess the reliability of historical reconstructions. As shown by the per-pixel analysis presented here, the 2014 dataset is already operating near the observational limit of the gap-filling approach; conducting a meaningful experiment requires automated cloud masking procedures and a multi-year, multi-mission dataset with genuine variability in observation density. These two lines of work define the roadmap for extending the methodology demonstrated here into a long-term monitoring system for Arctic vegetation phenology.

## Supplementary Information

Below is the link to the electronic supplementary material.

Supplementary Material 1 (DOCX 12.7 KB)

## Data Availability

The Landsat 8 imagery used in this study is freely available through the U.S. Geological Survey (USGS) Earth Explorer platform (https://earthexplorer.usgs.gov/). Field phenological observations and derived SOS estimates are available from the corresponding author upon reasonable request.
