# Supplementary material for: Can time-series of Landsat data be used to map the onset of growth at 78°N (central Svalbard)?
Source: Int J Biometeorol. 2026 Jul 22;70(8):220. doi: 10.1007/s00484-026-03276-4 (PMC13391796; doi:10.1007/s00484-026-03276-4)
Supplement: Supplementary file 1 — Supplementary Material 1 (DOCX 12.7 KB) [file 484_2026_3276_MOESM1_ESM.docx]

Table S1 Panel A: Pearson correlations (n = 225,339), Panel B: Regression model performance

| **A** |  | **SOS** | **Elevation** | **Slope** | **Distance** |
| --- | --- | --- | --- | --- | --- |
|  | SOS | 1.000 | 0.257*** | -0.093*** | 0.151*** |
|  | Elevation |  | 1.000 | 0.402*** | 0.188*** |
|  | Slope |  |  | 1.000 | 0.066*** |
|  | Distance |  |  |  | 1.000 |

| **B** |  |  | **Model** | **R²** | **RMSE (days)** |
| --- | --- | --- | --- | --- | --- |
|  |  |  | Elevation only | 0.066 | 17.0 |
|  |  |  | Elevation + Slope + Distance | 0.123 | 16.5 |
|  |  |  | Full model (with interactions) | 0.146 | 16.3 |

***p < 0.001
